# Pterostilbene prevents AKT-ERK axis-mediated polymerization of surface fibronectin on suspended lung cancer cells independently of apoptosis and suppresses metastasis

**DOI:** 10.1186/s13045-017-0441-z

**Published:** 2017-03-21

**Authors:** Ying-Jan Wang, Jing-Fang Lin, Li-Hsin Cheng, Wen-Tsan Chang, Ying-Hsien Kao, Ming-Min Chang, Bour-Jr Wang, Hung-Chi Cheng

**Affiliations:** 1 Department of Environmental and Occupational Health, National Cheng Kung University, 1 University Road, 70101 Tainan, Taiwan; 2 The Institute of Basic Medical Sciences, College of Medicine, National Cheng Kung University, 1 University Road, 70101 Tainan, Taiwan; 3 Department of Biochemistry and Molecular Biology, College of Medicine, National Cheng Kung University, 1 University Road, 70101 Tainan, Taiwan; 4 Department of Medical Research, E-Da Hospital, 82445 Kaohsiung, Taiwan; 5 Department of Biomedical Informatics, Asia University, Taichung, Taiwan; 6 Department of Medical Research, China Medical University Hospital, China Medical University, Taichung, Taiwan; 7 Graduate Institute of Clinical Medicine, Taipei Medical University, Taipei, Taiwan; 8 Department of Occupational and Environmental Medicine, National Cheng Kung University Hospital, Tainan, Taiwan; 9 Department of Cosmetic Science and Institute of Cosmetic Science, Chia Nan University of Pharmacy and Science, Tainan, Taiwan

**Keywords:** Fibronectin, Pericellular assembly, Pterostilbene, Pulmonary localization, PI3K/AKT/ERK signaling

## Abstract

**Background:**

Polymeric fibronectin (polyFN) assembled on suspended breast cancer cells is required for metastasis. Conceivably, drugs that target such polyFN may fight against cancer metastasis. While stilbene analogs trigger pro-apoptotic effect on attached cancer cells, whether they prevent polyFN assembly and metastasis of suspended cancer cells via an apoptosis-independent manner remains unexplored.

**Methods:**

We depleted suspended Lewis lung carcinoma (LLC) cells of polyFN by silencing the endogenous FN expression or pterostilbene (PS) to examine whether metastasis of lung cancer cells could thus be suppressed. We investigated whether PS regulates AKT-ERK signaling axis to suppress polyFN assembly in suspended LLC cells independently of apoptosis. We tested the therapeutic effects of orally administered PS against cancer metastasis.

**Results:**

Both FN-silencing and PS among the three stilbenoids indeed significantly reduced polyFN assembly and lung metastasis of suspended LLC cells in an apoptosis-independent manner. Mechanistically, PS-induced AKT phosphorylation (pAKT) and suppressed ERK phosphorylation (pERK) in suspended LLC cells, whereas pretreatment with a PI3K inhibitor, LY294002, effectively reduced pAKT, rescued pERK, and consequently reversed the PS-suppressed polyFN assembly on LLC cells; these pretreatment effects were then overturned by the ERK inhibitor U0126. Indeed, PS-suppressed lung metastasis was counteracted by LY294002, which was further overruled with U0126. Finally, we found that PS, when orally administered in experimental metastasis assays, both significantly prevented lung colonization and metastasis of LLC cells and reduced the already established tumor growth in the mouse lungs.

**Conclusions:**

PS suppressed AKT/ERK-regulated polyFN assembly on suspended LLC cells and pulmonary metastasis. PS possesses potency in both preventing and treating lung metastasis of lung cancer cells in apoptosis-independent and apoptosis-dependent manners, respectively.

**Electronic supplementary material:**

The online version of this article (doi:10.1186/s13045-017-0441-z) contains supplementary material, which is available to authorized users.

## Background

Lung cancer is the leading cause of cancer deaths worldwide [1, 2]. The majority of current cancer therapies pursue the goal of a better cytotoxic effect on cancer cells, aiming for tumorigenic suppression [3]. Unfortunately, the outcomes of these therapeutic strategies often ironically lead to increased mortality due either to the subsequent drug resistance and to metastatic recurrence or tumor cell dissemination within the circulation upon surgical removal of tumor tissues [4]. This issue has put scientists in a dilemma and has drawn worldwide attention. In complement with both primary tumor-targeted cytotoxic approaches and surgical strategies, effective inhibition of metastases by means of post-operational measures against circulating tumor cells (CTCs), even including those that are insensitive to cytotoxic therapies, may alternatively be an ideal anti-cancer adjuvant strategy [5, 6]. However, not enough attention has been devoted to developing this therapeutic modality against lung cancer.

Prior to metastasis, extracellular matrix (ECM) proteins contribute to the primary tumor or circulatory microenvironments that eventually facilitate efficient colonization of blood-borne tumor cells in distant organs [7, 8]. Tumor-associated ECM, including collagen and fibronectin (FN), is characterized by the polymerization of fibrillar components on tumor cell surfaces both in adherent and in suspended statuses [8–11]. During FN polymerization, disulfide-bonded dimer FN is polymerized into mature polymeric FN (polyFN) through a self-assembly process that is mediated by other covalent bonds than the disulfide bond [12]. Such polyFN is morphologically formed on suspended tumor cell surfaces as randomly distributed puncta [10, 11, 13]. The vascular arrest and metastasis of CTCs in the lungs are fundamentally mediated by the binding between endothelial dipeptidyl peptidase IV (DPP IV) and polyFN assembled on tumor cell surfaces [10, 13, 14]. It is the mature and irreducible polyFN that is assembled on suspended tumor cell surfaces remains on the well-top of SDS-PAGE stacking gel (GT) [10] and exposes the otherwise cryptic DPP IV-binding sites [13] mediates the binding of DPP IV and lung metastasis [10]. Clinically, FN expression has been identified as one of the prognostic biomarkers in CTCs of non-small cell lung cancer (NSCLC) and pancreatic cancer patients [15–17]. Consistently, FN expression in gastric cancer cells and invasive breast cancer cells has also been strongly correlated to poor prognoses and low survival rates [18, 19]. Thus, metastatic CTC-associated FN has a potential to serve as an anti-cancer therapeutic target [20]. Although an investigation into responsible signaling mechanisms has shown that protein kinase Cε is involved in the regulation of polyFN assembly on suspended tumor cells and consequently results in pulmonary metastasis [11], other participating signaling regulators remain largely elusive. A mechanistic elucidation of polyFN-depleting reagents in metastatic suppression may help unveil undiscovered pathways in regulating polyFN assembly.

Phytochemical agents have often been used as alternative medicine as an attempt to improve the traditional cancer therapeutic consequences [21]. Stilbenoids, hydorxylated derivatives of stilbene including resveratrol, oxyresveratrol, and rhapontigenin, are a group of natural compounds existing in ripened blueberries and grapes [22]. During the past decades, numerous studies have found that, due to structural homology, most stilbenoids possess similar biomodulatory functions including anti-oxidative and anti-inflammatory effects [23]. In addition, resveratrol displays anti-tumor activity [24]. Among the resveratrol analogs, pterostilbene (PS) has a higher bioavailability and is more lipophilic, leading to better oral absorption and cellular uptake [23]. Despite preclinical evidence revealing that these dietary compounds suppress lung tumorigenesis through triggering apoptosis in tumor cells [23], little is known about how or even whether or not they can effectively prohibit blood-borne tumor cells from colonizing the lungs by depleting the polyFN on their surfaces without causing apoptosis. Therefore, the purposes of this study were to validate the role of polyFN in the lung metastasis of Lewis lung cancer cells (LLCs), to examine the ability of stilbene derivatives to prevent polyFN assembly of suspended LLCs and suppress lung metastasis in both in vitro and in vivo settings, and to explore the underlying apoptosis-independent pathway responsible for the polyFN-depletion by stilbene derivatives. Our results clearly demonstrated that silencing endogenous FN expression in suspended LLCs significantly reduced their polyFN assembly and pulmonary metastasis. In line with these results, PS, among the three other stilbene derivatives, effectively suppressed polyFN assembly and inhibited the pulmonary metastasis of various suspended tumor cells, including LLCs, in an apoptosis-independent manner. Mechanistically, the polyFN-depletion by PS was mediated by a signaling pathway involving AKT/ERK cascade. Importantly, PS by oral gavage both significantly reduced pulmonary metastasis and growth of LLC cells in the lungs in the experimental metastasis models.

## Methods

### Reagents

The four stilbenoids (pterostilbene, resveratrol, oxyresveratrol, and rhapontigenin) were obtained from Sabinsa Corp. (East Windsor, NJ). The purity of four compounds was determined by high-performance liquid chromatography as higher than 98%. 3-(4,5-dimethylthiazol-2-yl)-2,5-diphenyltetrazolium bromide (MTT), CaCl_2_, HEPES, paraformaldehyde (PFA), and crystal violet from Sigma-Aldrich, Inc. (St. Louis, MO, USA) and Carboxyfluorescein succinimidyl ester (CFSE) and Hoechst 33258 were from Invitrogen (Carlsbad, CA, USA). ERK inhibitor U0126, PI3K inhibitor LY294002 (LY), and bovine serum albumin (BSA) were from Cyrusbioscience (Taipei, Taiwan). Monoclonal antibodies (mAbs) against ERK1/2, pERK1/2, AKT, pJNK, JNK, and pp38 were from Cell Signaling (Beverly, MA, USA). 6A3 mAb was home-made [10, 25]. Polyclonal antibodies (pAbs) against pAKT, p38, and GAPDH were from Biolegend (San Diego, CA, USA); anti-FN pAb was obtained from Sigma-Aldrich, Inc. (St. Louis, MO, USA). Annexin V-FAM apoptosis detection reagent was from LEADGENE (Tainan, Taiwan). Purified DPP IV ^(31–767)^ was prepared from lungs of Fischer 344 rats as described in our previous publication [26].

### Cell culture

LLC cell line (ATCC: CRL-1642) was from the American Type Culture Collection. CNS-1 cells used to generate CNS-1 TR50 cells and human metastatic lung cancer CL1-5 cells were generous gifts from Dr. Chun-I Sze [Department of Cell Biology and Anatomy, National Cheng Kung University (NCKU)] and Dr. Yi-Ching Wang (Department of Pharmacology, NCKU), respectively. All cell types were cultured in Dulbecco’s modified Eagle’s medium (DMEM) (Gibco BRL, Grand Island, NY) supplemented with 10% fetal bovine serum (FBS) (Gibco BRL, USA). End-over-end (EoE) suspension culture was performed as previously described [11]. Tissue culture plastic ware was either purchased from BD Falcon (Franklin Lakes, NJ, USA) or from Wuxi NEST Biotechnology Co., Ltd (Wuxi, Jiangsu, China).

### Generation of lentivirus and small hairpin RNA (shRNA) interference

The RNAi reagents for lentiviral vector system were from the National RNAi Core Facility supported by the National Research Program for Genomic Medicine Grants of NSC (NSC 100-2314-B-006-055). The mouse library is referred to as TRC-Mm 1.0. Individual clones are identified as shRNA TRCN00000231750 for SCR control, TRCN00000306574, and TRCN0000090371 for FN-silencing shFN#1 and shFN#2, respectively; and TRCN0000022936 and TRCN0000302357 for AKT-silencing shAKT#1 and shAKT#2, respectively. The lentiviruses for RNAi techniques were produced as previously described [27].

### Animals and in vivo metastasis assays

All experiments on mice were performed according to the guidelines of our institute (the Guide for Care and Use of Laboratory Animals, NCKU Medical College). Four-week-old male C57BL6 mice were acquired from the Animal Center of the NCKU Medical College and were housed by five per cage at 24 ± 2 °C and 50 ± 10% relative humidity and were subjected to a 12-hour light/12-hour dark cycle. In experimental metastasis assays [14], LLC cells were treated according to individual design and were intravenously injected into C57BL6 mice. The lung tissues were dissected and subjected to H&E staining for further histological examination on metastatic tumor nodules. For tumor colonization assays, similar treatments of LLC cells to those for experimental metastasis assays were labeled with 20 μM CFSE at 37 °C for 30 min prior to the intravenous injections. Mice were sacrificed 30–36 h after tumor cell injections. Mouse lungs were removed and subjected to lung vasculature perfusion with PBS. The images of lung-colonized CFSE-labeled LLC cells were taken by Multiphoton Confocal Microscope BX61WI (FV1000MPE; Olympus, Tokyo, Japan) and were subjected to quantifications with ImageJ software.

### Evaluation for the therapeutic effects of orally administered PS on cancer metastasis

C57BL6 mice orally received DMSO vehicle or 5 mg/kg of PS (twice/day) 2 h prior to intravenously inoculation of 5 × 10^5^ LLC cells that were recovered in 20% FBS/DMEM for 2 h at 37 °C and then the daily PS-feeding schedule was continued for two more days. Alternatively, PS-feeding was scheduled from the fifth day after LLC inoculation to the end of the experiment. Tumor nodules in the lungs of the sacrificed mice were photographed, counted, and subjected to H&E staining.

### Cell viability assays

The treated adherent LLC cells were subjected to MTT assays as previously described [28]. Alternatively, LLC cells seeded in 96-well plates were subjected to 5 μg/ml of UV-excited Hoechst 33258 dye that was used to stain the cell nuclei in live cells 5 h before each time point. At the end point of each experiment, 1 μg/ml of non-cell permeable propidium iodide (PI), together with Hoechst 33258 dye, was added into the wells to stain the dead or the damaged cells. Both Hoechst 33258 dye-positive and PI-positive LLC cells were imaged.

### FACS analysis for apoptotic LLC cells

The DMSO or PS-treated LLC cells either in adherent or suspended statuses were subjected to Annexin V-FAM and PI apoptosis detections for 20 min at room temperature according to the manufacturer’s instruction. After PFA fixation, apoptotic cells were quantified with FACS analysis (FACSCalibur, BD Biosciences, CA, USA) as previously described [11].

### Immunofluorescence staining

LLC cells in EoE suspension culture after various treatments were subjected to immunofluorescence staining for polyFN assemblies as previously described [11].

### Protein preparation and western blotting

Total LLC cellular protein lysates of EoE suspension culture were prepared as previously described [10]. ProteoJET™ Membrane Protein Extraction Kit (Thermo Inc., San Jose, CA, USA) was used to prepare cytosolic and membrane protein extracts according to manufacturer’s instruction. Protein samples were then subjected to SDS-PAGE, electrotransferation, and western immunoblotting (IB). The chemiluminescence images of IBs were developed with CyECL reagents (Cyrusbioscience, Taipei, Taiwan) according to the manufacturer’s instruction.

### Wound-healing assay

LLC EoE suspension cultured cells were treated with different concentrations of PS for 4 h and seeded onto 6-well plates. Confluent adherent cells were then carefully scratched to mimic a linear “wound” in monolayer cells using a plastic pipette tip. After 48 h, the number of the cells that migrated into the initial cell-free zone was counted under microscope, based on the wounding edge at time zero.

### Transwell migration assay and matrigel invasion assay

The same LLC EoE suspension cultured cells as that for wound healing assays were seeded onto transwell inserts (8-μm pore, BD Falcon, Franklin Lakes, NJ, USA) in which 20-μg Matrigel (BD Falcon) was absent for migration assays and precoated for invasion assays as previously described [14].

### Image quantification methods

ImageJ software that was developed at the National Institutes of Health and is freely available from internet was used to quantify all the images of immunofluorescence staining and IB blotting. The statistical analyses for the images were applied in at least three repeated experiments.

### Statistical analysis

Data derived from at least three separate experiments are expressed as the mean ± SD. Statistical significance was determined by using Student’s *t* test for comparison between the means or one-way analysis of variance with post hoc Dunnett’s test [29]. Differences were considered to be significant when *p* value <0.05 (*), *p* < 0.01(**), or *p* < 0.001(***).

## Results

### PolyFN assembly on suspended LLC cells is required for pulmonary metastasis

**Fig. 1:**
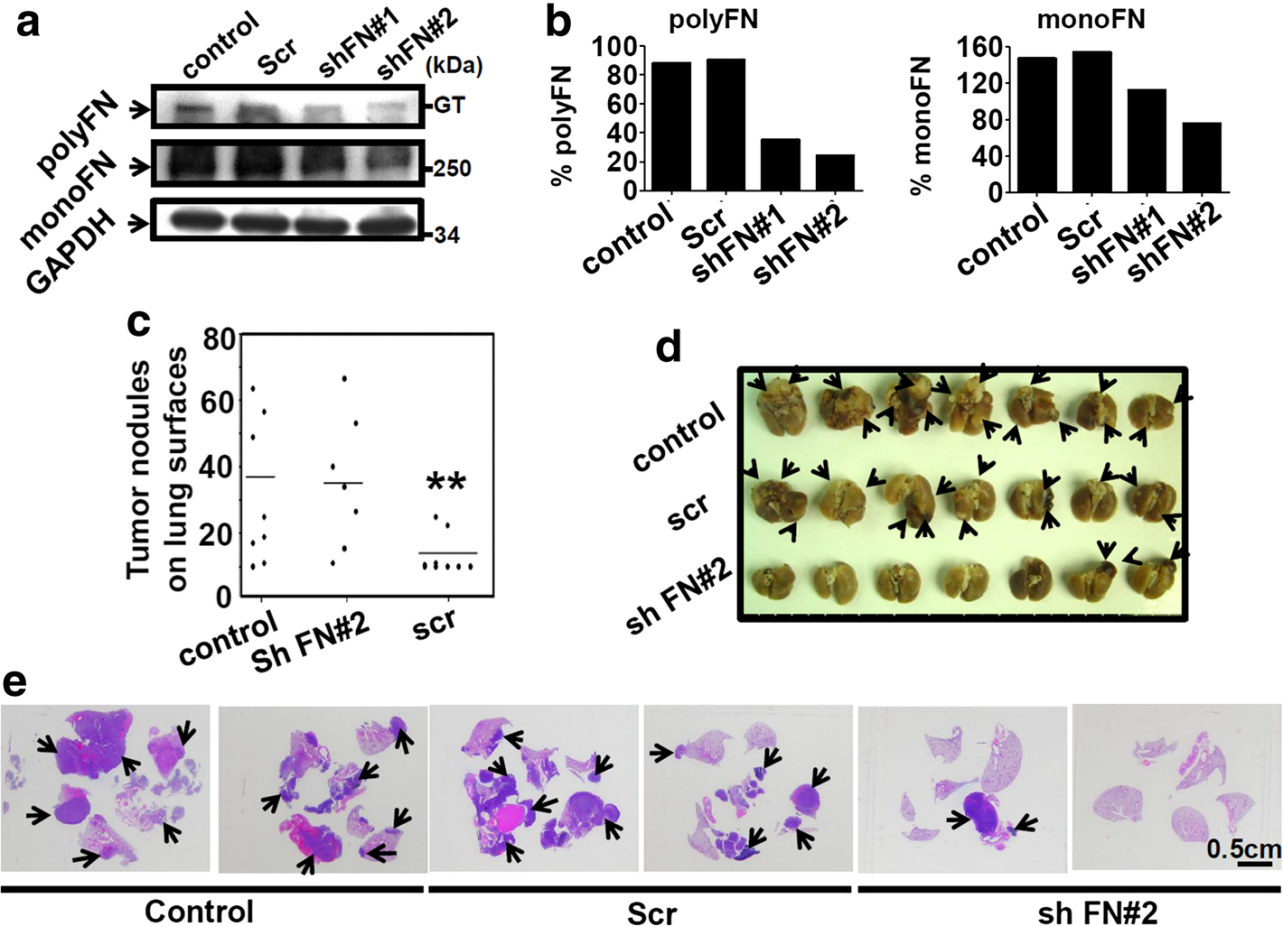
Endogenous FN expression is required for polyFN assembly on suspended LLC cells, which promotes lung metastasis. **a** The IB was probed with anti-FN pAb (*upper* and *middle panel*) or anti-GAPDH (*lower*) as input control for the whole cell lysates of LLC cells alone (*control*) or LLC cells expressing Scr shRNA (*Scr*) or FN shRNAs (*shFN#1* and *shFN#2*) that were resolved in SDS-PAGE under reducing conditions. The polyFN means polymeric FN form (*upper panel*) and monoFN monomeric FN form (*middle panel*). GT means well-top of stacking gel. **b** The quantifications of expression levels of polyFN (% polyFN; *left panel*) and monoFN (% monoFN) by ImageJ software as normalized with expression levels of GAPDH. **c** Tumor nodule numbers on mouse lung surfaces were counted, and **d** mouse lungs were taken from mice that intravenously received LLC cells as described in (**a**) upon mouse sacrifices. **e** H&E staining for tumor nodules in mouse lungs in (**d**). Two representative lungs of each group were demonstrated. Note: *arrow heads* depict tumor nodules in the lungs

We employed shRNA-mediated FN gene silencing to investigate how endogenous FN expression contributes to the polyFN assembly on suspended lung cancer cells. Since covalently cross linkage in addition to disulfide bond occurs during the maturation of polyFN [10], that FN shRNAs (shFN#1 and shFN#2) treatments significantly decreased polyFN in suspended LLC cells under reducing conditions as represented by FN on GT (Fig. 1a, b) suggested the requirement of endogenous FN expression for FN polymerization during matrix assembly. Next, we demonstrated the polyFN puncta on suspended control and scramble shRNA (Scr), but not shFN#2, LLC cells (Additional file 1: Figure S1a; upper panel). The quantifications of polyFN puncta (Additional file 1: Figure S1a; lower panel) reconfirmed that the essential role of endogenously synthesize FN in polyFN assembly on suspended LLCs. Importantly, the FN-silencing did not result in significant growth difference from control and Scr LLC cells (Additional file 1: Figure S1b), suggesting that depletion of polyFN on suspended LLC cells does not affect cell viability, negating the possibility that the metastatic consequences are indirectly influenced by the death and/or prohibited the growth of polyFN-depleted LLC cells.

Next, we examined the role of polyFN on suspended LLC cells in pulmonary metastasis. We found that the averaged ratio of lung weight over body weight (LW:BW) (Additional file 1: Figure S1c, d) and lung tumor nodule numbers (Fig. 1c, d) upon mouse sacrifices was significantly decreased for mice intravenously receiving shFN#2 LLC cells as compared to those receiving control or Scr LLC cells. Histological observation revealed that the tumor nodules present in the mouse lungs of both control and Scr groups, but not the shFN#2 group, were quantitatively numerous with diverse nodule sizes (Fig. 1e and Additional file 1: Figure S1e). These results clearly supported that polyFN assembly is required for pulmonary metastasis of circulating tumor cells, and depletion of polyFN on suspended tumor cells may be a useful polyFN-targeted anti-metastatic strategy.

### PS is among the other stilbenoids that are most potent in depleting suspended tumor cells of polyFN by interfering transportation of FN across plasma membrane

**Fig. 2:**
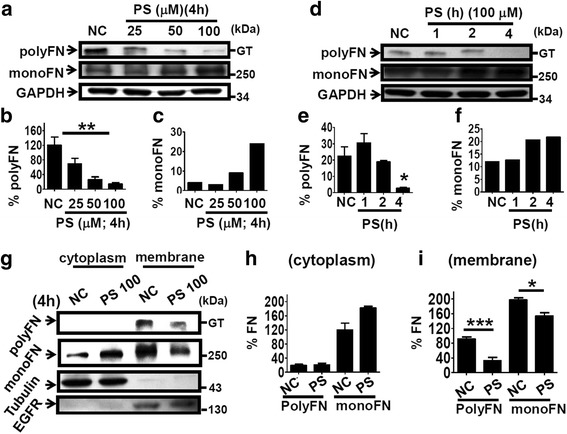
PS depletes suspended LLC cells of polyFN by interfering transportation of FN across plasma membrane. **a** IBs were probed with anti-FN pAb for lysates of LLC cells treated without or with various concentrations of PS for 4 h in suspension as indicated to reveal polyFN and monoFN expressions and anti-GAPDH mAb for the normalization purposes. Quantifications of polyFN (**b**) and of monoFN (**c**) that were normalized by GAPDH levels in (**a**). **d**–**f** Similar IBs and quantifications for the polyFN and monoFN as those in (**a**)–(**c**) of the lysates of suspended LLC cells treated with 100 μM of PS for different time points. **g** IBs were probed for polyFN, monoFN, tubulin as a marker for the cytoplasm fraction, and EGFR as a marker for the cell membrane fraction prepared from lysates of suspended LLC cells treated without or with 100 μM of PS. Quantifications of polyFN and monoFN in the cytoplasmic fractions (**h**) and in the cell membrane fraction (**i**)

We next tested four structurally related stilbenoids including resveratrol, oxyresveratrol, rhapontigenin, and PS, for their effects on polyFN-depletion from suspended LLC cells. PS was the most potent suppressor (Additional file 1: Figure S2a) to deplete LLC cells of polyFN in a dose- and time-dependent manner (Fig. 2a–f and Additional file 1: Figure S2b). Fluorescence visualization confirmed the prominent effect of PS on the polyFN-depletion (Additional file 1: Figure S2c). In addition to LLC cells, PS also significantly depleted the polyFN of suspended CL1-5 cells isolated from the tumor tissues of a human non-small cell lung cancer patient [14] (Additional file 1: Figure S2d) and suspended rat FN^high^-CNS-1 glioblastoma cells derived from a paclitaxol-resistant parental CNS-1cell line (Additional file 1: Figure S2e), suggesting that the polyFN-depletion effect of PS is not merely specific to suspended LLC cells and may be more widely applied to various metastatic and even chemo-resistant cancer types for therapeutic purposes.

Interestingly, the monoFN, unlike the effect of FN-silencing (Fig. 1b; right panel), was increased upon treatment of PS (Fig. 2a, c, d, f), suggesting that the diminished polyFN by PS is due to the prevention of monoFN from being polymerized and assembled into polyFN. We further investigated the underlying molecular mechanism. Fractionation of plasma membrane and non-membrane (cytoplasm) proteins from the PS-treated suspended tumor cells revealed that PS markedly reduced polyFN and, to a lesser extent, monoFN in plasma membrane, but significantly increased monoFN in the cytoplasm compartments (Fig.2g–i), suggesting that the suppression of polyFN by PS (Fig. 2a, c, d, f and Additional file 1: Figure S2) is mainly due to the inhibition of intracellular monoFN being transported onto the extracellular plasma membrane of tumor cells and subsequent polymerization into polyFN.

### Apoptosis does not contribute to polyFN-depletion from suspended LLC cells by PS

**Fig. 3:**
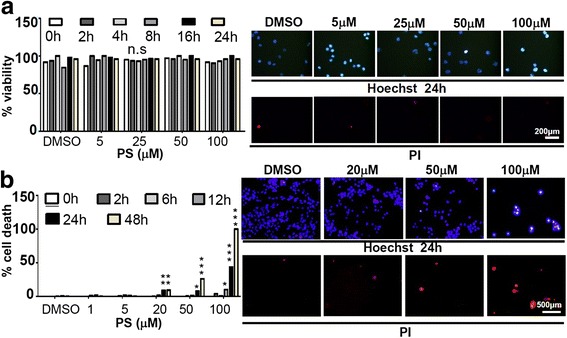
PS triggers apoptosis of LLC cells in adherent, but not suspended, status. **a** Suspended LLC cells treated with DMSO or various concentrations of PS in a time course (0–24 h) were stained with Hoechst and incubated with PI at each time point as indicated in centrifugation tubes. % cell viability was calculated as 1-PI^high^ cell number/ Hoechst^high^ cell number (*left panel*). *Right panel*: representative images showing the co-staining of Hoechst and PI in PS-treated suspended cells at the 24-h time point. **b** Adherent LLC cells were similarly treated with DMSO or PS and stained as in (**a**﻿) within the culture dishes. Cell death % was calculated as PI^high^ cell number/ Hoechst^high^ cell number (left panel). *Right panel*: representative images showing the co-staining of Hoechst and PI in PS-treated adherent cells at the 24-h time point

Since PS has long been known to be capable of inducing apoptosis of adherent tumor cells [23], it is possible that the depletion of polyFN on suspended LLC cells by PS was a consequence of tumor cell apoptosis. To test this possibility, we compared the apoptotic effects of PS on suspended and adherent LLC cells in a time-course study. While PS did not affect LLC viability in suspension even at a concentration as high as 100 μM for 24 h (Fig. 3a and Additional file 1: Figure S3), it significantly induced apoptosis of adherent LLC cells in a dose-dependent manner (Fig. 3b and Additional file 1: Figure S4). To firmly ensure that the PS-pretreatment does not influence the long-term viability of suspended LLC cells, we reseeded the vehicle- or PS-pretreated suspended LLC cells on the culture dishes and showed no difference for the cell growth and migratory activities (Additional file 1: Figure S5). Altogether, these results suggested that polyFN-depletion from suspended LLC cells by PS is not indirectly caused by apoptosis.

### PS effectively inhibits pulmonary metastasis of LLC cells

**Fig. 4:**
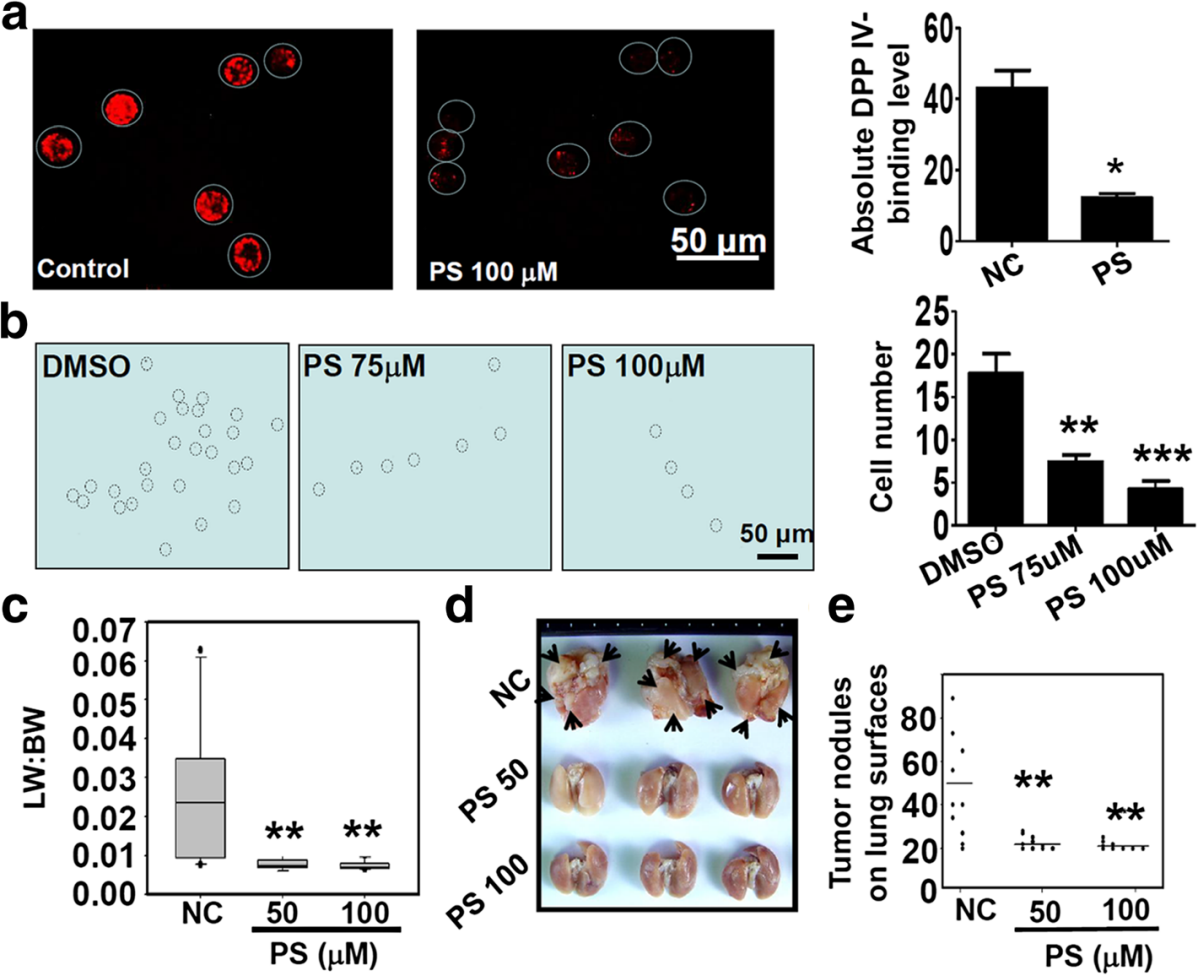
PS effectively prevents LLC cells from binding to DPP IV and colonizing and metastasizing to the lungs. **a** Suspended LLC cells treated with DMSO (*control*) or 100 μM PS were subjected to the binding of DPP IV ^(31–767)^ that was derived from full-length DPP IV and retained the polyFN-binding ability. The cells were then subjected to IF staining using 6A3 mAb and phycoerythrin-conjugated goat anti-mouse antibody. The averaged red fluorescence intensities (absolute DPP IV-binding levels) of representative cell images as circled with *white dashed lines* (*left two panels*) were quantified in the right panel. **b** Representative images (*left three panels*) and quantifications (*right panel*) of mouse lung-colonized suspended LLC cells that were treated with DMSO, 75 μM or 100 μM PS. *Circled dots* with *dashed lines* represent the colonized LLC cells labeled with CFSE dye as illustrated with ImageJ software. **c** Lung weight/body weight (LW:BW) of the lungs in mice intravenously receiving LLC cells treated with DMSO (*control*) or two concentrations of PS upon mouse sacrifices. Mouse lungs (**d**) were taken and tumor nodule numbers (**e**) on lung surfaces from mice as described in (**c**) were counted upon mouse sacrifices. *Black arrow* heads depict tumor nodules in the lungs

Since polyFN is required for endothelial DPP IV binding and lung metastasis of suspended LLC cells (Fig. 1a) [10], we next tested whether PS suppresses lung metastasis. We first demonstrated that treating suspended LLC cells with PS inhibited the binding of a soluble DPP IV ^(31–767)^ peptide possessing FN-binding ability [26] to polyFN (Fig. 4a), entertaining the possibility that PS could prevent circulatory tumor cells from adhering to the lung vasculature. We then tracked prelabeled individual LLC cell suspension and assessed the initial extravasation. Indeed, the ability of PS-treated LLC cells to colonize and to extravasate the mouse lungs was drastically reduced in a dose-dependent manner (Fig. 4b). We next examined whether PS reduces lung metastasis of suspended LLCs. The LW:BW ratios (Fig. 4c and Additional file 1: Figure S6a) and lung tumor nodules (Fig. 4d, e and Additional file 1: Figure S6b, c) were significantly decreased in PS-treated groups compared to the NC group. These results clearly demonstrated that PS effectively prevented polyFN assembly on LLC cells, reduced the lung colonization, and inhibited lung metastasis. Treating suspended LLC cells with PS at non-cytotoxic doses neither affected their motility as revealed by wound healing assays (Additional file 1: Figure S5), trans-well migration assays (Additional file 1: Figure S7a) nor their invasiveness as revealed by matrigel invasion assays (Additional file 1: Figure S7b), suggesting that the in vivo anti-metastatic effect of PS is not attributed to the suppression of migratory/invasive abilities of LLC cells. Nevertheless, these data did not exclude possibilities that PS inhibits cancer metastasis through polyFN-independent mechanisms [21–23].

### PS suppresses the polyFN assembly of suspended LLC cells by interfering with the AKT/ERK signaling pathway

**Fig. 5:**
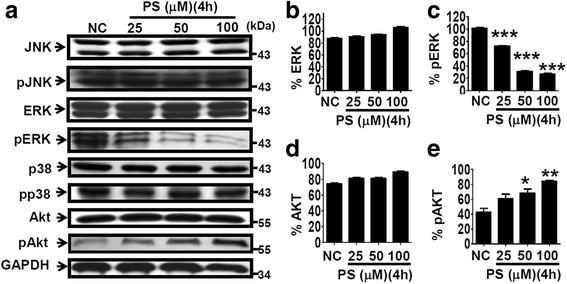
PS dose-dependently increases AKT phosphorylation and reduces ERK phosphorylation of suspended LLC cells. **a** IBs were probed for various signaling proteins, and their phosphorylation statuses as indicated in the lysates of suspended LLC cells were treated without or with various concentrations of PS for 4 h at 37 °C. Quantifications of ERK (**b**), pERK (**c**), AKT (**d**), and pAKT (**e**) were normalized by GAPDH levels in (**a**)

**Fig. 6:**
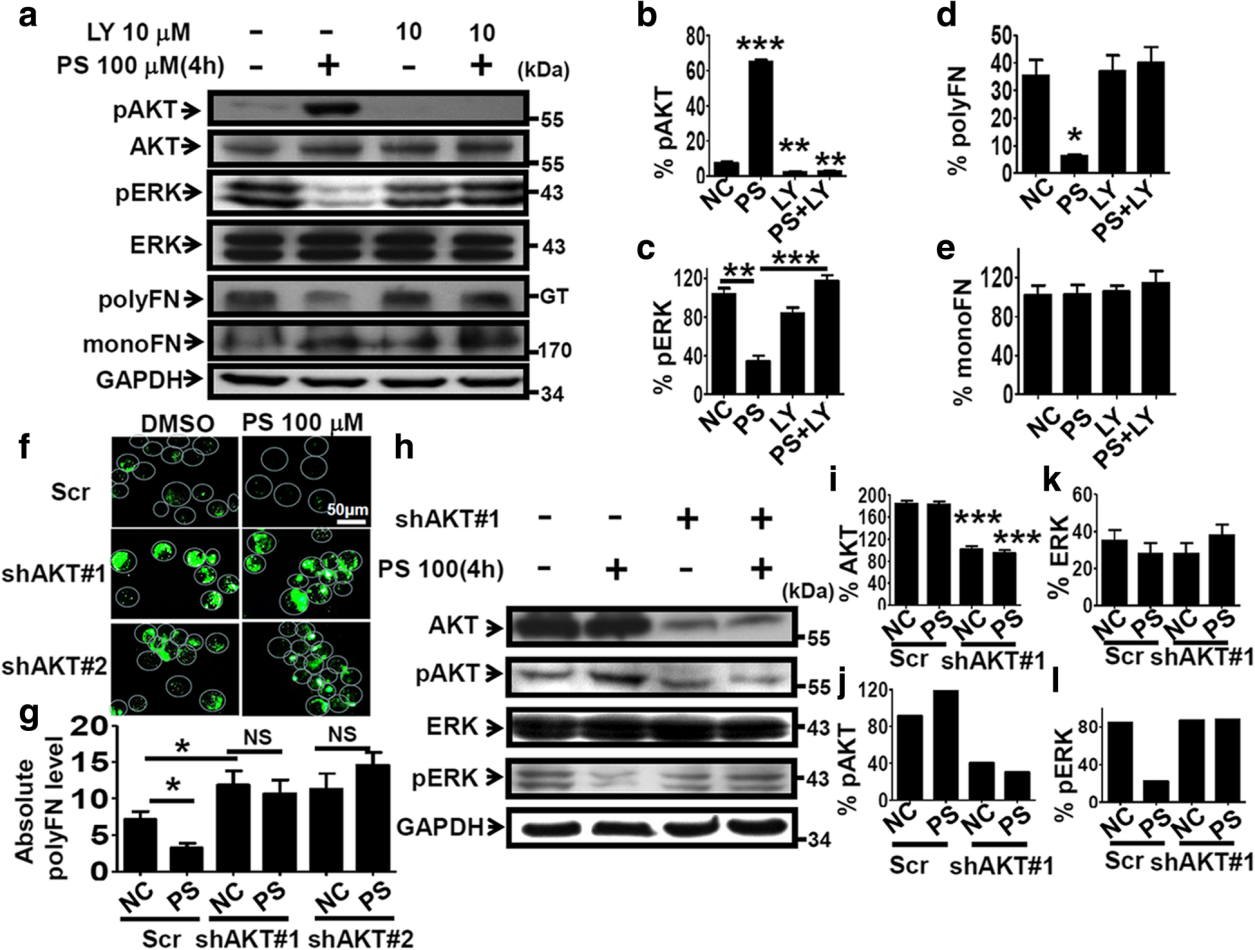
PS inhibits pericellular FN polymerization via triggering AKT phosphorylation which reduces ERK phosphorylation. **a** IBs were probed for AKT phosphorylation, ERK phosphorylation, polyFN, and monoFN in lysates of suspended LLC cells treated without or with 100 μM of PS, 10 μM of PI3K inhibitor LY294002 (LY) or mixture of both for 4 h in suspension. Quantifications of pAKT (**b**), pERK (**c**), polyFN (**d**), and of monoFN (**e**) that were normalized by GAPDH levels in the IBs in (**a**). **f** Representative images of IF staining for the polyFN assemblies on suspended LLC cells transfected with Scr or shAKT#1 and 2 in the absence or presence of 100 μM of PS for 4 h. **g** Quantifications of polyFN levels on LLC cells as the treatment in (**f**). **h** The same IBs as in (**a**) for lysates of suspended LLC cells transfected with Scr shRNA or shAKT#1 in the absence or presence of 100 μm of PS for 4 h. Quantifications of AKT (**i**), pAKT (**j**), ERK (**k**), and pERK (**l**) were normalized by GAPDH levels in the IBs in (**h**)

**Fig. 7:**
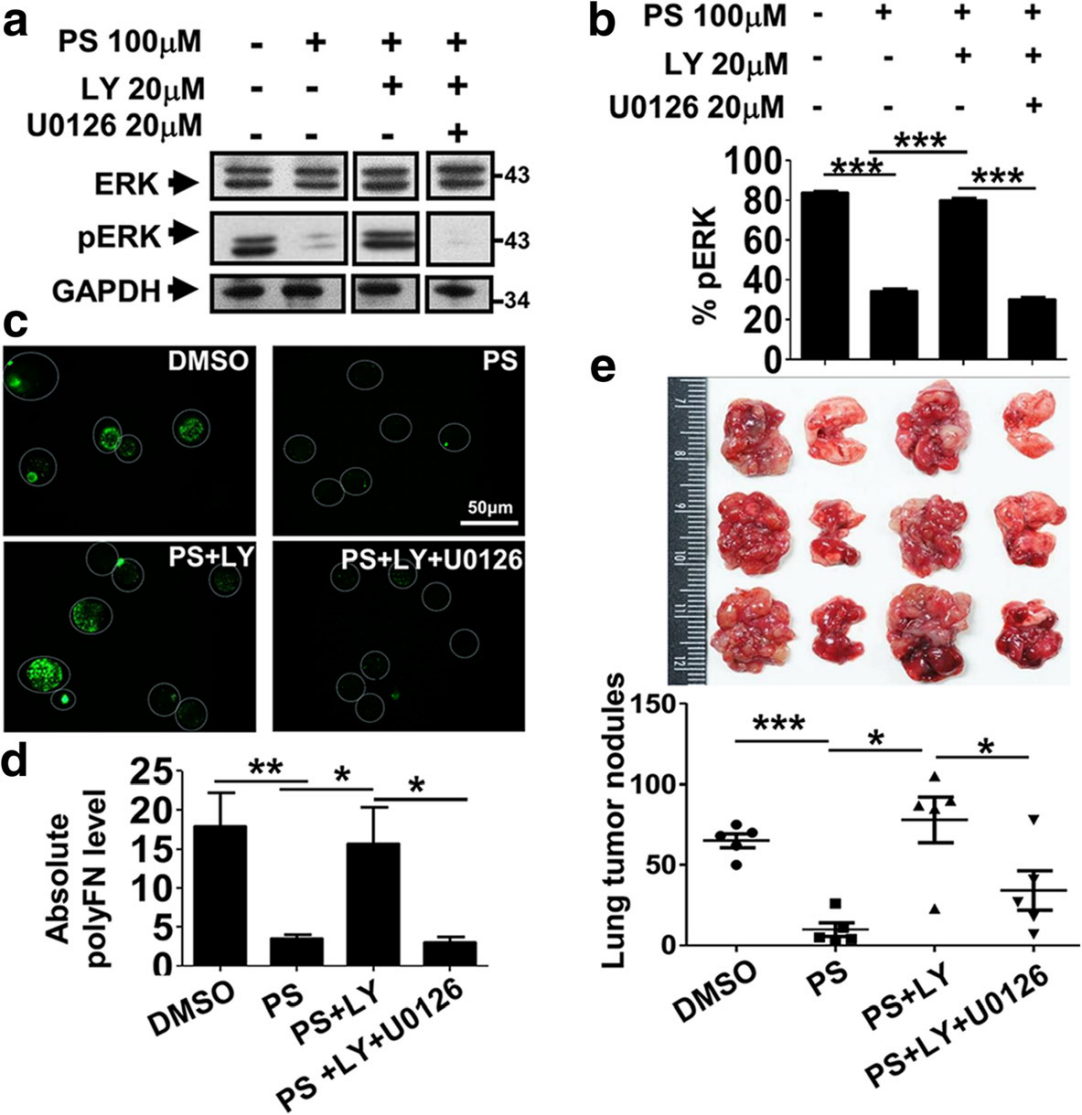
The activated PI3K/AKT-induced ERK inactivation mediates the inhibitory effect of PS on polyFN polymerization on and mouse lung metastasis of suspended LLC cells. **a** IBs were probed for total ERK, pERK, and GAPDH in lysates of suspended LLC cells treated without or with 100 μM of PS, PS + 20 μM of PI3K inhibitor LY or PS + LY+ 20 μM of ERK inhibitor U0126 for 4 h in suspension. Note: Images of the same proteins were trimmed from the same IB gels. **b** Quantifications of pERK that was normalized by GAPDH levels in the IBs in (**a**). **c** Representative images of IF staining for the polyFN assemblies on suspended LLC cells that were treated similarly as described in (**a**). The positions of tumor cells were circled with *white dashed lines*. **d** Quantifications of polyFN levels on LLC cells that were treated as in (**c**). **e** Mouse lungs (*upper panel*) were taken, and tumor nodule numbers (*lower panel*) on lung surfaces from mice bearing the intravenously inoculated LLC cells that were treated with DMSO, PS, PS + LY or PS + LY + U0126 as described in (**a**) were counted upon mouse sacrifices

We continued to ask how PS affects the signaling that regulates the polyFN assembly on suspended LLCs. Phosphorylation of intracellular proteins, including PI3K/AKT and MAPK signaling pathways, has been deemed an essential protein modification that facilitates tumor progression and metastasis [30]. Therefore, we examined the modulatory effects of PS on the phosphorylation of PI3K/AKT, JNK, p38, and ERK in suspended LLC cells. We found that 4 h of PS-treatment significantly increased AKT phosphorylation (pAKT) and concomitantly decreased ERK phosphorylation (pERK) in suspended LLC cells in a dose-dependent manner (Fig. 5a–e), whereas no difference was observed in the phosphorylation statuses of JNK and p38 (Additional file 1: Figure S8a–d). These results were different from what have been found for adherent cells [22] and attached LLC cells (Additional file 1: Figure S8e–g) and raised the possibility that PS-activated PI3K/AKT signaling inhibits ERK phosphorylation [31], thereby interrupting the polyFN assembly on suspended LLCs. LY294002 (LY), a common specific inhibitor of PI3K that directly regulates pAKT, and AKT shRNAs (shAKT#1 and shAKT#2) (Additional file 1: Figure S8i–j) were used to test this possibility. We showed that pretreatment with 10 μM LY effectively blocked the PS-induced pAKT in LLC cells (Fig. 6a, b and Additional file 1: Figure S9c). Meanwhile, it completely recovered the PS-decreased pERK (Fig. 6a, c and Additional file 1: Figure S9d) and polyFN levels (Fig. 6a, d, e, and Additional file 1: Figure S9a, b), substantiating that PS-inhibited ERK activity and subsequently polyFN assembly are both mediated by the elevated pAKT in LLC cells. In line with the effect of LY (Fig. 6a–e), immunofluorescent staining results confirmed the preventive effects of shAKT#1 and #2 on the assembly of polyFN puncta in the PS-treated LLC cells (Additional file 1: Figure S9e, f and Fig. 6f, g). Moreover, the requirement of PS-promoted pAKT for the lowered pERK was reconfirmed by transfecting the LLC cells with shAKT#1 prior to the PS-treatment (Fig. 6h–l). We next employed an ERK inhibitor U0126 in the PS-treated LLC cells (PS alone) and found that the LY-rescued pERK (Fig. 7a, b) and polyFN assemblies (PS + LY) (Fig. 7c, d) were re-abolished (PS + LY + U0126) (Fig. 7a–d), confirming that PS-depleted polyFN assembly is mediated by the AKT-induced inactivation of ERK in LLC cells. Strikingly, the LY-recovered LLC metastasis that was inhibited by PS was drastically re-suppressed by U0126 (Fig 7e and Additional file 1: Figure S10). These findings further corroborated that the PI3K/AKT/ERK signaling axis is involved in the PS-suppressed polyFN assembly and metastasis of circulating tumor cells.

### Orally administered PS suppresses lung metastasis of LLC cells

**Fig. 8:**
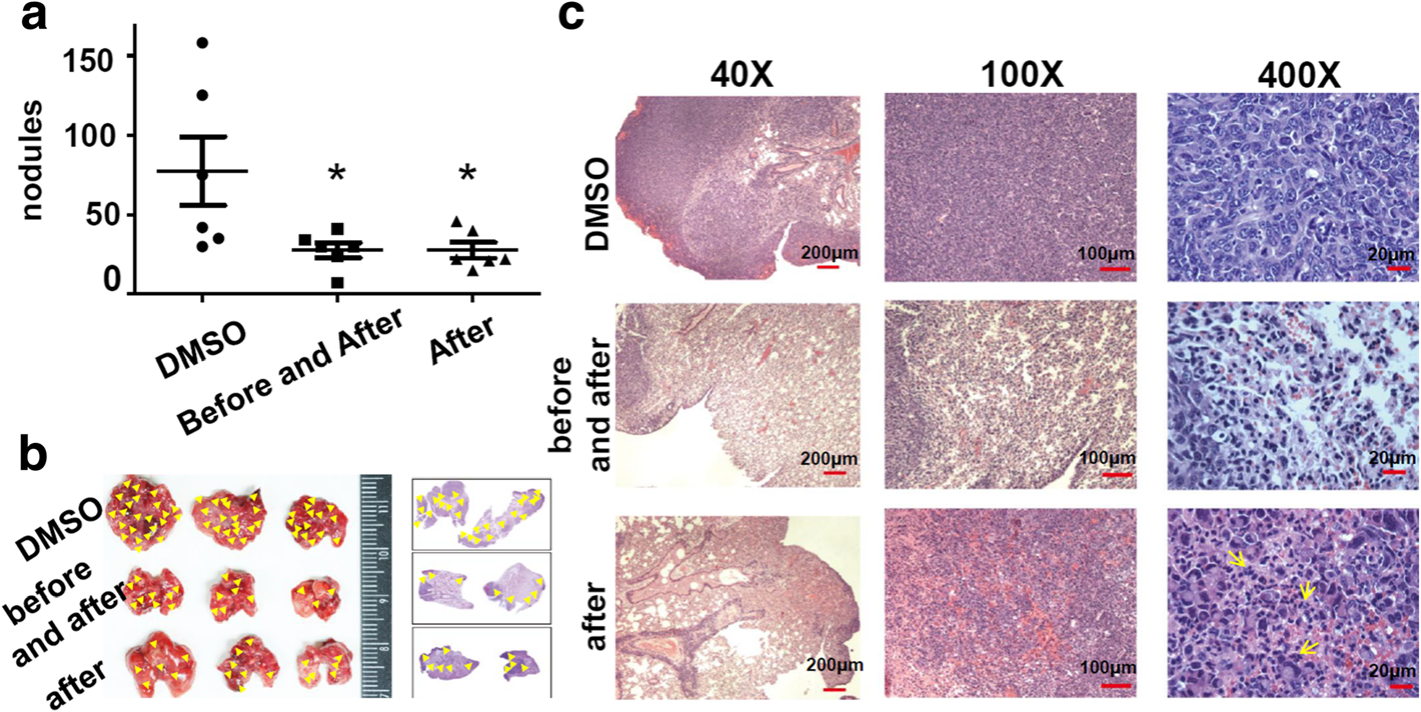
Oral administration of PS prevents lung-colonization of intravenously inoculated tumor cells and suppresses the growth of already established tumor nodules in the lungs. **a** Tumor nodules in the lungs of the sacrificed mice who orally received DMSO vehicle or 5 mg/kg of PS (twice/day) from before to after (before and after) or 5 days after (after) intravenously inoculation of 5 × 10^5^ LLC cells that were recovered in 20% FBS/DMEM for 2 h at 37 °C. The mice were sacrificed 30 days after tumor injection. **b** Representative mouse lungs with tumor nodules and their H&E staining from the mice as described in (**a**). **c** Microscopies of the H&E staining of mouse lungs as described in (**b**). Note: * means *p* value <0.05. *Red lines* in the magnitudes of 40×, 100×, and 400× mean 200, 100, and 20 μm, respectively. *Arrow heads* mean the tumor nodules in the lungs. *Arrows* mean the apparent apoptotic tumor cells with fragmented DNAs

**Fig. 9:**
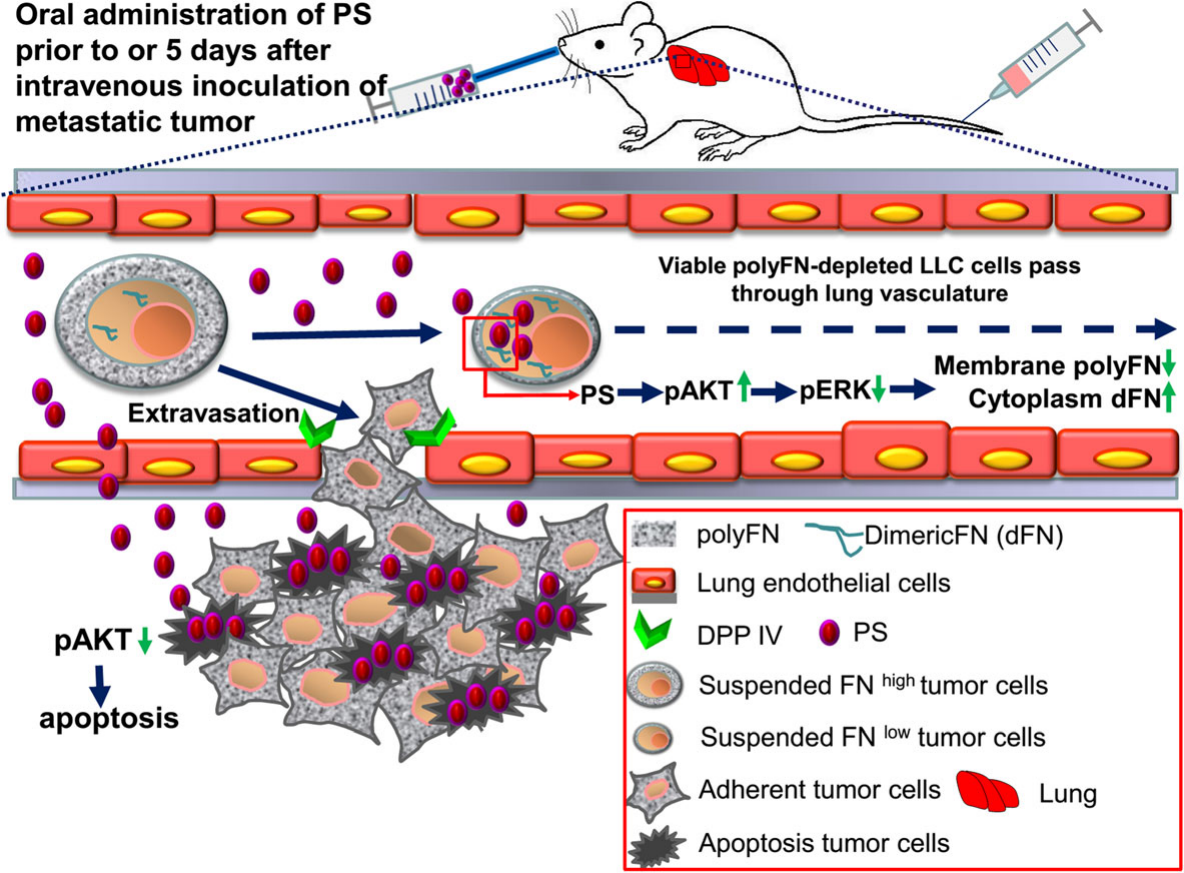
Schematic illustration for the roles of orally administered PS in metastatic suppressions either via polyFN-depletion and prevention of lung colonization of circulating tumor cells or via triggering apoptotic effect on adherent tumor cells that have already been established in the lungs

Since PS is one of the phytochemical agents that have often been orally used as an alternative medicine for tumor treatment, we tested whether tumor metastasis could also be prevented via oral administration of PS. We found that lung metastasis of LLC cells was significantly reduced when PS was orally administered 24 h prior to the intravenous inoculation of LLC cells and was given twice a day for two more days to maintain the PS in the circulation (Fig. 8a–c). These results indicated that the prevention of tumor cell extravasation within the circulation of the lungs could be accomplished by orally giving PS. Interestingly, the already-established metastasis was also decreased when PS was orally given 5 days after LLC inoculation, and the treatment was continued twice a day until the mice were sacrificed (Fig. 8a–c), suggesting that PS evokes cytotoxic effects on the extravasated tumor cells in adherent status in the lungs. Indeed, this notion was supported by the histopathological findings in which more inflammatory responses were often observed (Fig. 8c). Altogether, our findings promisingly shed light on PS that might serve as an alternative phytochemical medicine with preventive as well as therapeutic values for late stage cancer patients due to its multiple anti-metastatic effects (Fig. 9).

## Discussion

FN has been regarded as an anti-cancer therapeutic target [15, 20]. We have shown that polyFN assembled on blood-borne cancer cells facilitates cancer metastasis in the lungs [11, 13, 25]. Consistently, anti-polyFN and anti-DPPIV strategies have been verified in cancer metastatic prevention [13, 25]. Using RNAi techniques, the present study is the first to employ an experimental metastasis model to reveal that depleting circulating lung tumor cells of polyFN could serve as a therapeutic strategy against metastasis. Furthermore, our findings that PS, a well-known phytochemical, significantly inhibited the polyFN assembly and lung metastasis of blood-borne lung cancer cells strongly suggest that the anti-FN strategy for cancer patients is potentially applicable in metastatic prevention and prognostic improvement.

Endogenous synthesis of FN has been found to be required for the polyFN assembly on breast cancer cells [11]. Here, we also showed the same requirement for the lung cancer cells to assemble polyFN (Fig. 1). However, the preventive effect of PS on the translocation of intracellular dimeric FN onto the extracellular compartment of LLC cells (Fig. 2) suggested that the mechanism involved in the depletion of polyFN by PS is not due to the transcriptional inhibition of FN biosynthesis, but instead to the prevention of cell surface localization of dimeric FN to be later polymerized into polyFN. Consistent with our results, it has been shown that the newly synthesized FN is a disulfide-bonded dimer which is relatively deoxycholate (DOC)-soluble and can be easily reduced into monoFN by reducing reagents, whereas the polyFN in its polymeric status becomes DOC-insoluble and non-reducible [32]. A possible explanation for such phenomenon is that, during FN translocation between the rough endoplasmic reticulum (RER) and Golgi complexes, monoFN could be dimerized merely through two C-terminal disulfide bonds [32], whereas plasma membrane-located dimeric FN could further be polymerized through intermolecular disulfide bonds [32], and covalent bonds most likely triggered by cell surface tissue transglutaminase not present within the RER/Golgi compartments [33].

It has been recognized that anoikis is triggered through a significant inactivation of the PI3K/AKT survival signal pathway when normal cells lose their adhesions to the surrounding ECM and commit to apoptosis [34]. However, the blood-borne metastatic tumor cells evolve to be anoikis-resistant, and the PI3K/AKT survival signals persist in the absence of ECM anchorages [34, 35]. Consistently, we showed that the PS-treated lung tumor cells in suspension, where the sustained AKT signal was further activated, failed to colonize and metastasize to the lungs within the circulation but survived well (Figs. 2, 3, 4, 5, 6, 7), which was apparently distinct from those effects of PS on adherent cells as manifested by the AKT inactivation and apoptosis [23]. Due to the entirely distinct extracellular microenvironments between adherent and circulating tumor cells [6], the signaling pathways in the tumor cells within such distinct microenvironments conceivably also differ even when responding to the same drug treatment. Since AKT activity has been reported to be involved in promoting multiple tumor functionalities in addition to triggering cell proliferation and survival [36], it is expected that PS suppresses the polyFN of suspended tumor cells via the AKT/ERK signaling axis independently of apoptotic inhibition. It has been proposed that PS may cause apoptosis in adherent cells by directly binding to one of the various intracellular target molecules, including CYP1A1, CYP1A2, CYP1B1, and metastasis-associated protein 1 (MAP-1) [37, 38]. In line with this concept, PS might bind to the same downstream target molecules to suppress metastasis of blood-borne tumor cells in an apoptosis-dispensable manner [39, 40].

Combination therapies have recently prevailed over single drug treatment, particularly for cancer patients suffering late stages of malignancy [41]. The essence of most combinatory drugs for cancer therapeutics is to prevent cancer cell resistance to apoptosis-inducing drugs [41]. However, cancer cells still tend to develop resistance to apoptotic assaults and become even more metastatic [42, 43]. It is desired that combinatory drugs alternatively attack other later progression steps than the original tumor cell growth [44, 45] to complement traditional chemotherapeutics or to fight with chemoresistance. Importantly, preferred drugs are desired to exert multiple functions by concomitantly inducing apoptosis of tumor cells in the solid tumor tissues and triggering apoptosis-independent mechanisms to prevent metastasis of CTCs. Here, we found that PS fulfilled such a desire for multiple functionalities (Fig. 8). In line with such fulfillment, PS has been found capable of evoking multiple signaling pathways in mediating apoptotic-dependent and apoptotic-independent activities [38, 46], making itself a suitable anti-metastatic therapeutic regimen to target circulating tumor cells [6, 47]. Moreover, PS has better anti-tumor potency than resveratrol due to its superior bioavailability [23, 48]. Furthermore, recent in vivo safety analyses further revealed that PS, as a major natural product in our daily foods, does no harm to mice [49] and humans [50], suggesting that PS may be a useful therapeutic agent by either systemic or oral administration for the prevention and treatment of lung metastasis. In this regard, comparable oral administrative dosages of PS as used in our studies have also been tested in mice for the efficacies of anti-tumor growth and anti-oxidant activity [23, 51].

## Conclusions

Our studies demonstrated a dramatic inhibition of FN polymerization on suspended LLC cells receiving shFN and PS treatments. Both AKT- and ERK-regulated signaling pathways are involved in the PS-suppressed polyFN assembly on suspended cancer cells. Intravenous administration of the FN-silenced or PS-treated tumor cells in turn resulted in a reduction of metastatic nodules in mouse lungs. When PS was orally given, it possibly exerted multiple functions by concomitantly triggering apoptosis-independent mechanisms to prevent CTC metastasis and inducing apoptosis in tumor cells already established in the lungs. Altogether, we propose that both the induction of apoptosis in solid tumor cells and blockade of polyFN assembly on circulating tumor cells by PS are potent therapeutic strategies for the prevention and treatment of lung cancer metastasis (Fig. 9).

## Additional file

Additional file 1: 
**Figure S1.** The reduced lung metastasis of FN-silenced suspended LLC cells is not due to inhibited cell proliferation and to the lowered mouse body weights. **Figure S2.** PS is among other stilbenoids the best inhibitor against periFN assembly of suspended mouse lung cancer cell line LLC, human lung cancer cell line CL1-5, and rat glioblastoma cell line CNS-1 TR50. **Figure S3.** Viabilities of PS-treated suspended LLC cells. Suspended LLC cells were similarly treated with DMSO or PS for up to 48 h, stained, and analyzed as described in methodology. **Figure S4.** Viabilities of PS-treated adherent LLC cells. **Figure S5.** PS-pretreatment for LLC cells does not impair their growth and migratory activities upon reseeding onto dishes. **Figure S6.** PS inhibits periFN assembly and metastasis of LLC cells. **Figure S7.** Migration and invasion activities of the reseeded LLC cells that were pretreated with various concentrations of PS in suspension. **Figure S8.** PS has no effect on activities of JNK and p38. **Figure S9.** Effects of PI3K inhibitor and shAKT on polyFN assemblies and expression levels of AKT and ERK. **Figure S10.** LW:BWs from each mouse bearing intravenously inoculated suspended LLC cells that were treated with DMSO, PS, PS+LY, or PS+LY+U0126 as described in Fig. 8a. (PDF 1380 kb)

## Abbreviations

BSA: Bovine serum albumin
CFSE: Carboxyfluorescein succinimidyl ester
CTCs: Circulating tumor cells
DOC: Deoxycholate
DPP IV: Dipeptidyl peptidase IV
ECM: Extracellular matrix
EoE: End-over-end
FACS: Fluorescence activated cell sorting
IB: Immunoblotting
LLC: Lewis lung carcinoma
LY: LY294002
mAbs: Monoclonal antibodies
monoFN: Monomeric FN
MTT: 3-(4,5-dimethylthiazol-2-yl)-2,5-diphenyltetrazolium bromide
NCKU: National Cheng Kung University
NSCLC: Non-small cell lung cancer
pAbs: Polyclonal antibodies
pAKT: AKT phosphorylation
pERK: ERK phosphorylation
PFA: Paraformaldehyde
polyFN: Polymeric FN on suspended tumor cell surfaces
PS: Pterostilbene
RER: Rough endoplasmic reticulum
RNAi: RNA interference
shAKT: AKT shRNAs
shFN: FN shRNAs
shRNA: Small hairpin RNA
