# Supplementary material for: Pterostilbene prevents AKT-ERK axis-mediated polymerization of surface fibronectin on suspended lung cancer cells independently of apoptosis and suppresses metastasis
Source: J Hematol Oncol. 2017 Mar 21;10:72. doi: 10.1186/s13045-017-0441-z (PMC5361845; doi:10.1186/s13045-017-0441-z)
Supplement: Additional file 1: — Figure S1. The reduced lung metastasis of FN-silenced suspended LLC cells is not due to inhibited cell proliferation and to the lowered mouse body weights. Figure S2. PS is among other stilbenoids the best inhibitor against periFN assembly of suspended mouse lung cancer cell line LLC, human lung cancer cell line CL1-5, and rat glioblastoma cell line CNS-1 TR50. Figure S3. Viabilities of PS-treated suspended LLC cells. Suspended LLC cells were similarly treated with DMSO or PS for up to 48 h, stained, and analyzed as described in methodology. Figure S4. Viabilities of PS-treated adherent LLC cells. Figure S5. PS-pretreatment for LLC cells does not impair their growth and migratory activities upon reseeding onto dishes. Figure S6. PS inhibits periFN assembly and metastasis of LLC cells. Figure S7. Migration and invasion activities of the reseeded LLC cells that were pretreated with various concentrations of PS in suspension. Figure S8. PS has no effect on activities of JNK and p38. Figure S9. Effects of PI3K inhibitor and shAKT on polyFN assemblies and expression levels of AKT and ERK. Figure S10. LW:BWs from each mouse bearing intravenously inoculated suspended LLC cells that were treated with DMSO, PS, PS+LY, or PS+LY+U0126 as described in Fig. 8a. (PDF 1380 kb) [file 13045_2017_441_MOESM1_ESM.pdf]

Additional file

Introduction

The detailed information in each additional figure is as followed:

1. For Fig. S1: a LLC cells expressing shRNAs were IF stained with anti-FN pAb (*upper panel*) and were quantified for the polyFN assemblies (*lower panel*). Inset: higher magnitudes of the representative images of cells with or without polyFN assemblies. Absolute polyFN level means absolute fluorescence represented by pixel value. b LLC cell proliferation assays for five days. c Body weights (*BWs*) of mice after intravenously receiving the three LLC cells as described in Fig. 1a were measured for 30 days. d Lung weight/body weight (*LW:BW*) of the mouse lungs in (c) upon mouse sacrifices. e H&E staining for tumor nodules in the lungs of mice intravenously receiving LLC cells expressing control (*non-treatment*), Scr, or sh FN. Note: arrow heads depict tumor nodules in the lungs.
2. For Fig. S2: **a** Quantifications for the inhibitory effects of stilbenoids on polyFN of LLC cells that were treated with 50 μM for 2 h. Note: NC means cells treated with vehicle DMSO. **b** Quantifications for the dose-dependent inhibitory effect of PS on polyFN of LLC cells in a time course with various time points. **c** LLC cells treated with DMSO (control), 50 , or100 μM PS for 4 h were IF stained with anti-FN pAb (*left panel*) and quantified (*right panel*) for the polyFN assemblies. Inset: higher magnitudes of the representative images of cells with or without periFN assemblies. Absolute polyFN level means absolute fluorescence represented by pixel value. **d** CL1-5 and **e** CNS-1 TR50 cells treated with vehicle DMSO or 100 uM PS for 4 h were IF stained with anti-FN pAb (*left panel*) and quantified (*right panel*) for the polyFN assemblies. *Cyan arrows* depict the remnants of polyFN upon PS treatment. Note: The positions of tumor cells within (d) and (e) were circled with white dashed lines.
3. For Fig. S4: Adherent LLC cells treated with DMSO or various concentrations of PS and stained with Hoechst for different times as indicated were incubated with PI and stained with Annexin V. Cells were then resuspended and subjected to FACS analyses at each time point.
4. For Fig. S5: Suspended LLC cells pretreated with DMSO (NC) or various concentrations of PS for 4 h in the presence of 20% FBS were reseeded onto dishes. The wounds were made (*before*) prior to wound-healing assays for 24 h (*after 24 h*).
5. For Fig. S6: **a** The body weights (*BWs*) of mice intravenously receiving LLC cells treated with DMSO (*control*) or two concentrations of PS as indicated were measured at various time points for 30 days. **b** H&E staining for tumor nodules in mouse lungs

taken after mouse sacrifices. **c** Microscopy of representative images of H&E staining for tumor nodules as shown in (b). Note: arrows depict tumor nodules in the lungs.

6. For Fig. S7: The same treatments for LLC cells as in Fig. S5 were subjected to transwell migration assays (**a)** or to matrigel invasion assays (**b)**. Representative images of LLC cells that migrated through membrane in the transwell in (a) or through membrane underneath the matrigel in the transwell in (b) are shown in left panel and % migrated cells were counted as illustrated in right panel.

7. For Fig. S8: Quantifications of total JNK **(a)**, pJNK **(b)**, total p38 **(c)**, and pp38 **(d)** that were normalized with GAPDH expression levels in Fig. 4J. **e** IB probed with anti-AKT pAb (*upper panel*) or anti-GAPDH (*middle panel*) as input control for the whole cell lysates of LLC cells alone (*control*) or LLC cells expressing Scr shRNA (*Scr*) or AKT shRNAs (*shAKT#1 and shAKT#2*). IB of AKT expression levels were quantified with Image J (lower panel). Quantification for the IB of pAKT (**f**) and pERK **(g)** were normalized with AKT and ERK protein levels, respectively, with Image J.

8. For Fig. S9: **a** Representative images of IF staining for the polyFN assemblies on suspended LLC cells treated with DMSO, 100 μM PS, 10 μM LY, or LY+PS for 4 h. **b** Quantifications of polyFN levels on LLC cells as the treatment in (a). Quantifications of AKT (**c**) and ERK (**d**) that were normalized by GAPDH levels in the IBs in Figure 6a. **e** IB probed with anti-AKT pAb (*upper panel*) or anti-GAPDH (*lower panel*) as input control for the whole cell lysates of LLC cells alone (control) or LLC cells expressing Scr shRNA (*Scr*) or AKT shRNAs (*shAKT#1 and shAKT#2*). **f** IB of AKT expression levels were quantified with Image J.

Legends

Figure S1. The reduced lung metastasis of FN-silenced suspended LLC cells is not due to inhibited cell proliferation and to the lowered mouse body weights. **Figure S2.** PS is among other stilbenoids the best inhibitor against periFN assembly of suspended mouse lung cancer cell line LLC, human lung cancer cell line CL1-5, and rat glioblastoma cell line CNS-1 TR50. **Figure S3.** Viabilities of PS-treated suspended LLC cells. Suspended LLC cells were similarly treated with DMSO or PS for up to 48 h, stained, and analyzed as described in methodology. **Figure S4.** Viabilities of PS-treated adherent LLC cells. **Figure S5.** PS-pretreatment for LLC cells does not impair their growth and migratory activities upon reseeding onto dishes. **Figure S6.** PS inhibits periFN assembly and metastasis of LLC cells. **Figure S7.** Migration and invasion activities of the reseeded LLC cells that were pretreated with various concentrations of PS in suspension. **Figure S8.** PS has no effect on activities of JNK

and p38. **Figure S9.** Effects of PI3K inhibitor and shAKT on polyFN assemblies and expression levels of AKT and ERK. **Figure S10.** LW:BWs from each mouse bearing intravenously inoculated suspended LLC cells that were treated with DMSO, PS, PS+LY, or PS+LY+U0126 as described in Fig 8A.

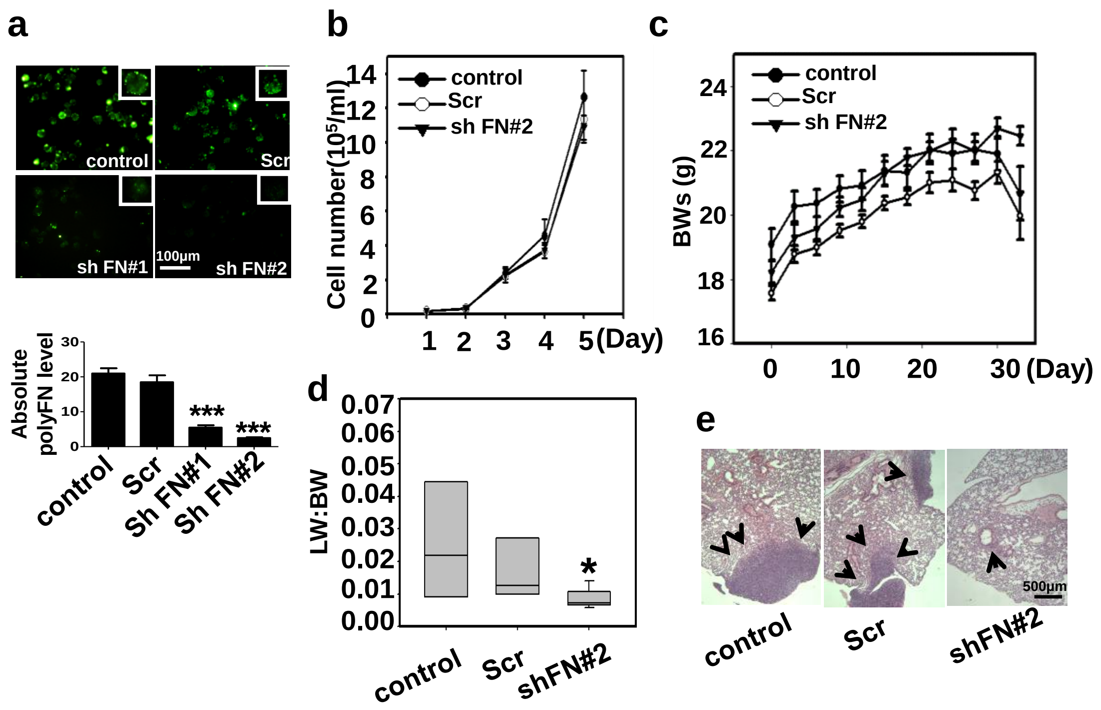

Figure S1. The reduced lung metastasis of FN-silenced suspended LLC cells is not due to inhibited cell proliferation and to the lowered mouse body weights.

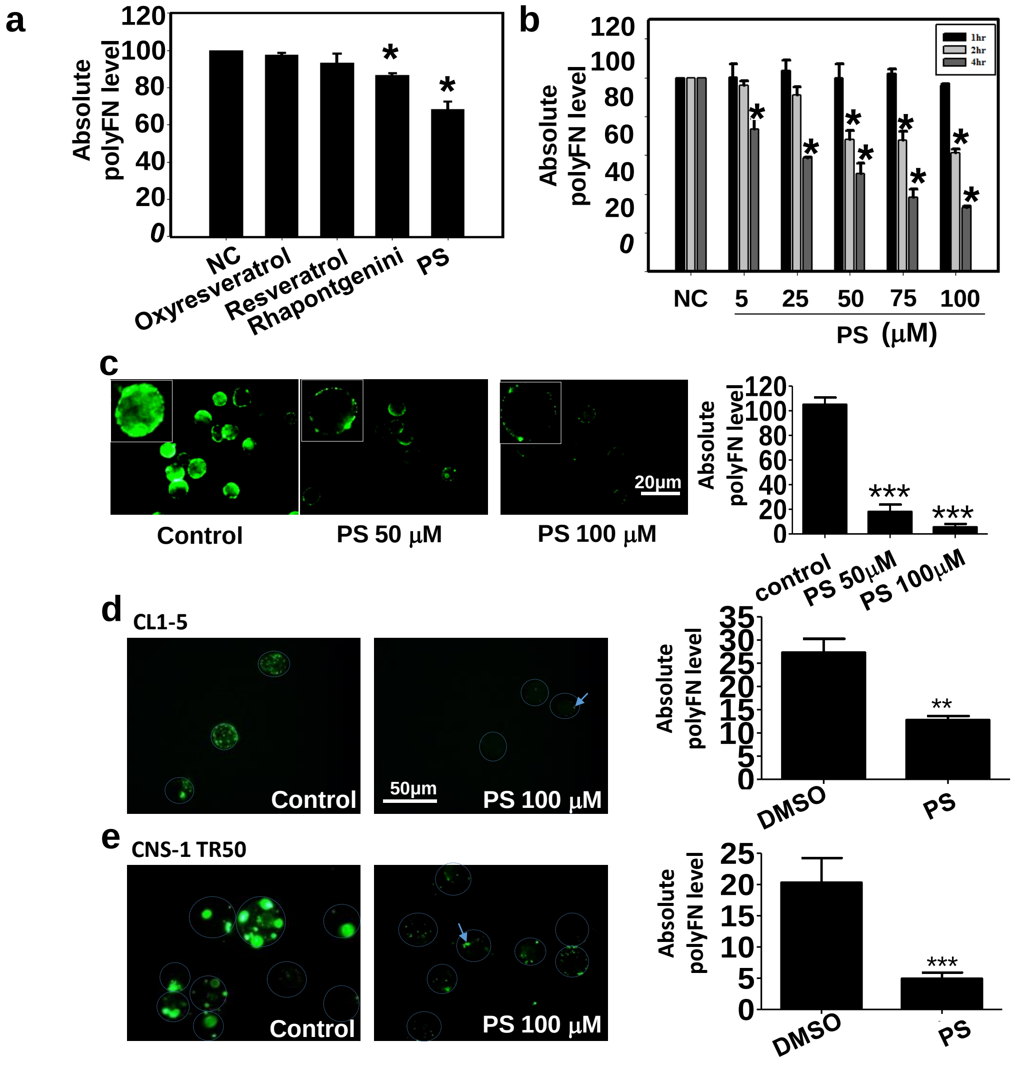

Figure S2. **PS is among other stilbenoids the best inhibitor against periFN assembly of suspended mouse lung cancer cell line LLC, human lung cancer cell line CL1-5, and rat glioblastoma cell line CNS-1 TR50.**

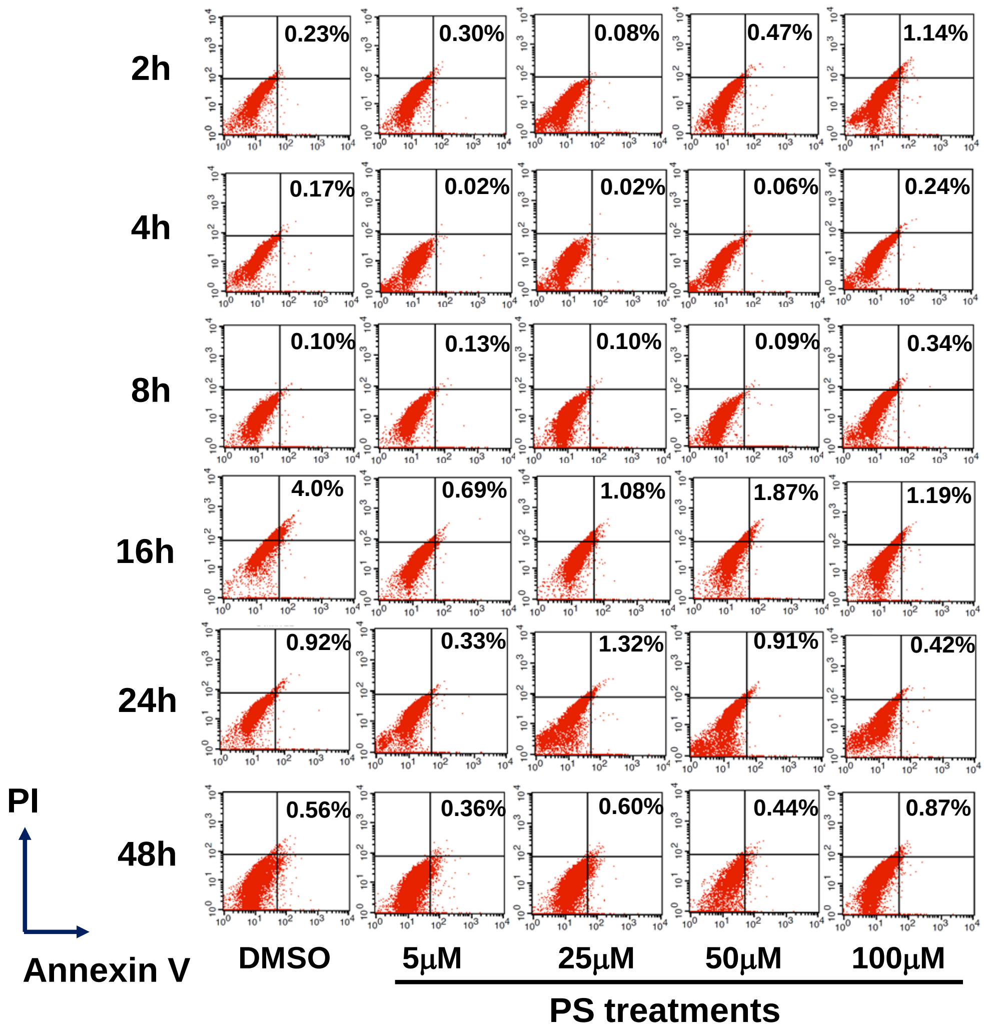

Figure S3. Viabilities of PS-treated suspended LLC cells. Suspended LLC cells were similarly treated with DMSO or PS for up to 48 h, stained, and analyzed as described in methodology.

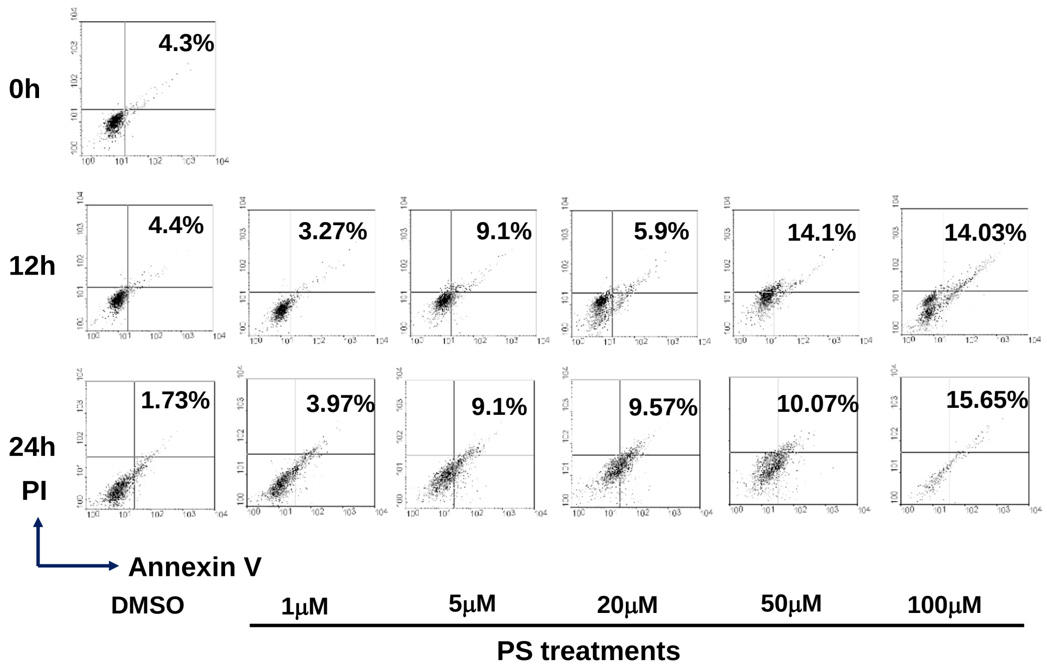

Figure S4. Viabilities of PS-treated adherent LLC cells.

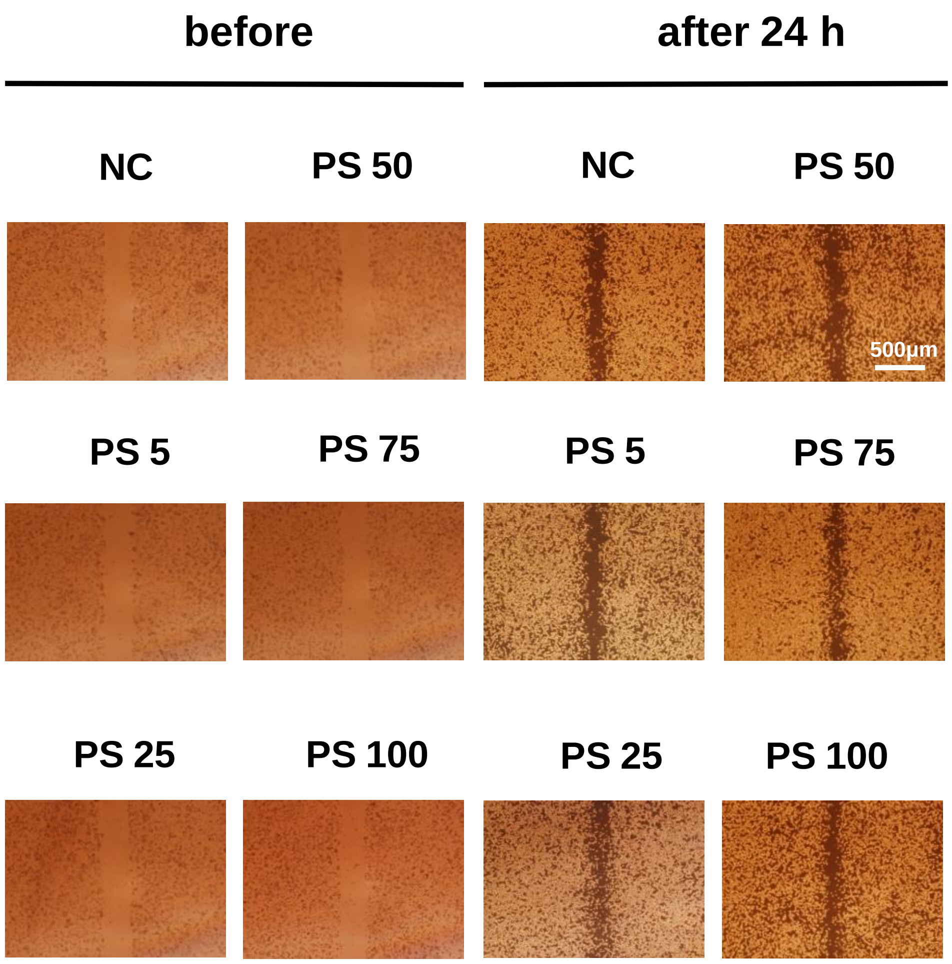

Figure S5. PS-pretreatment for LLC cells does not impair their growth and migratory activities upon reseeding onto dishes.

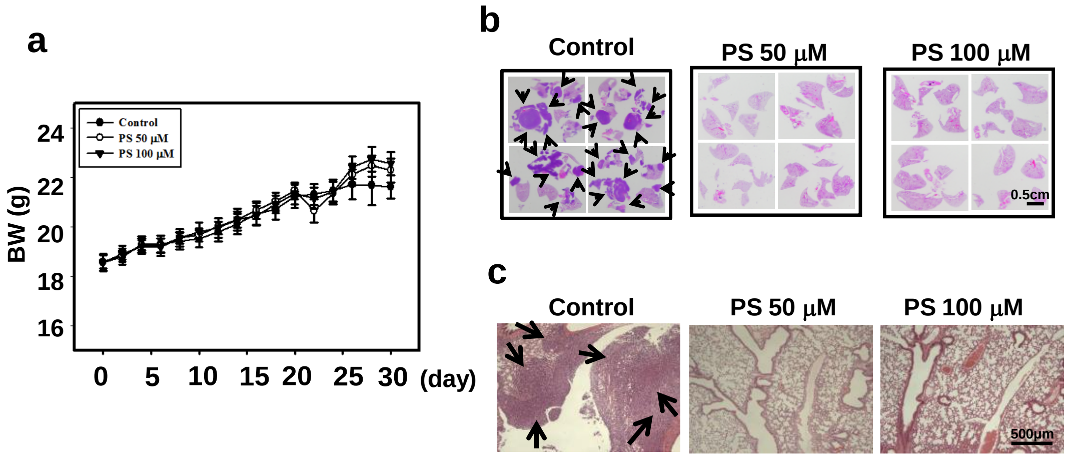

Figure S6. PS inhibits periFN assembly and metastasis of LLC cells.

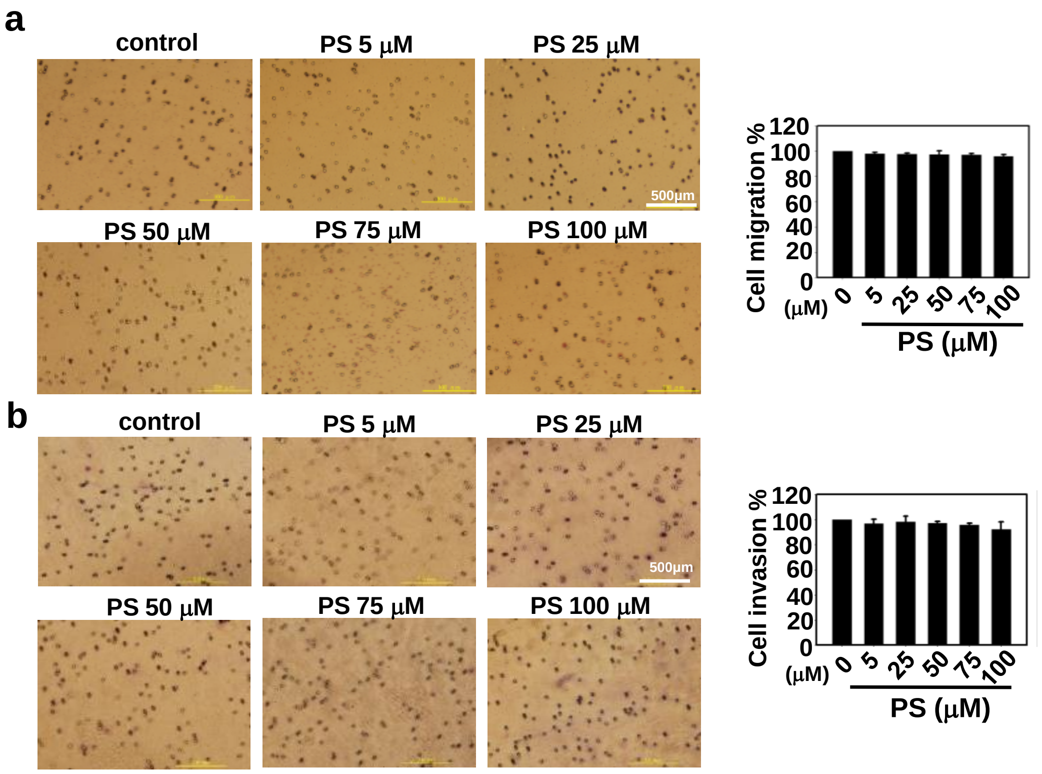

Figure S7. Migration and invasion activities of the reseeded LLC cells that were pretreated with various concentrations of PS in suspension.

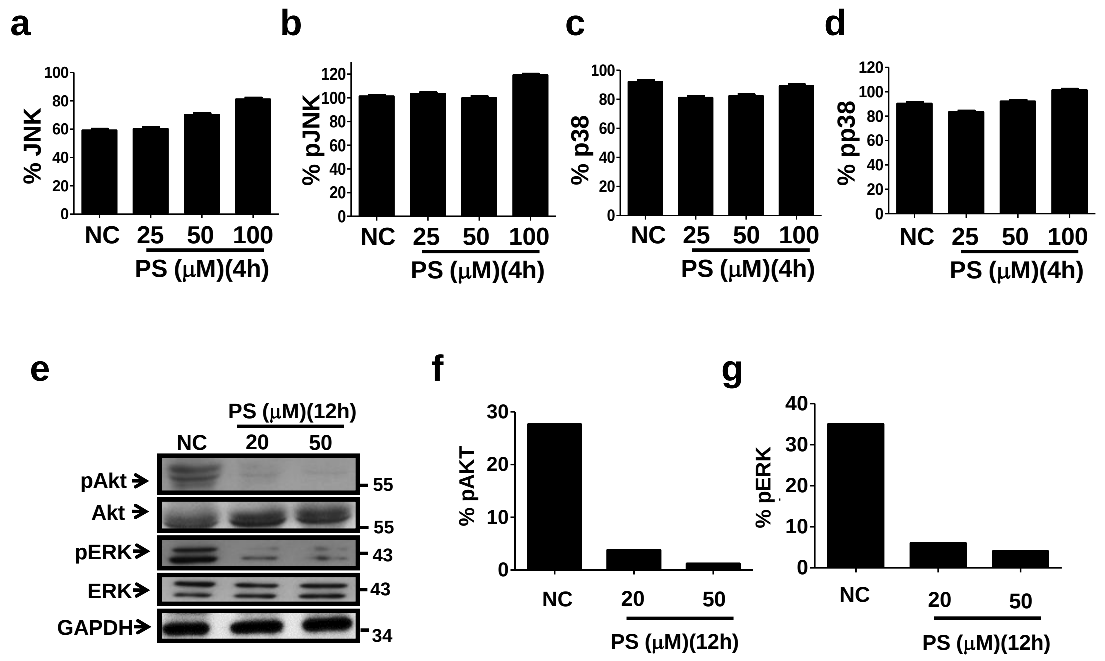

Figure S8. PS has no effect on activities of JNK and p38.

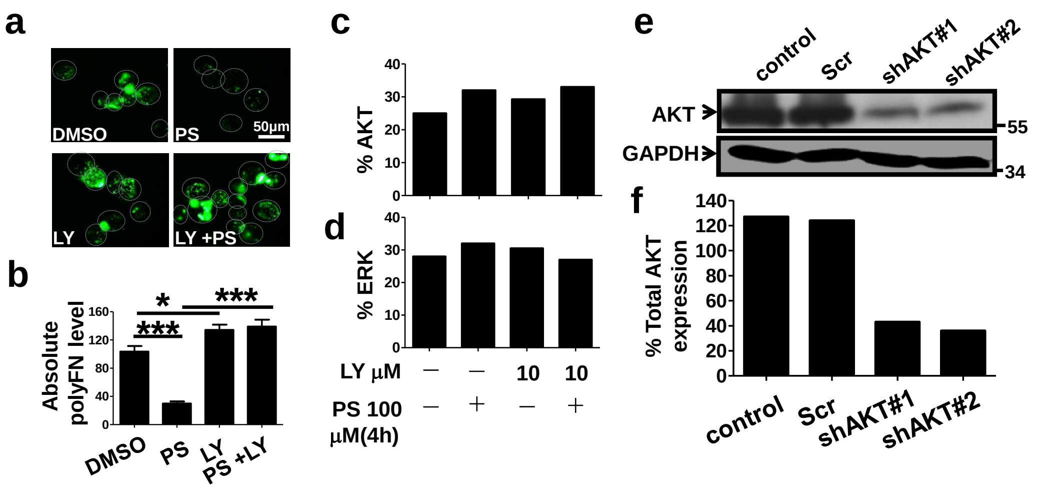

Figure S9. Effects of PI3K inhibitor and shAKT on polyFN assemblies and expression levels of AKT and ERK.

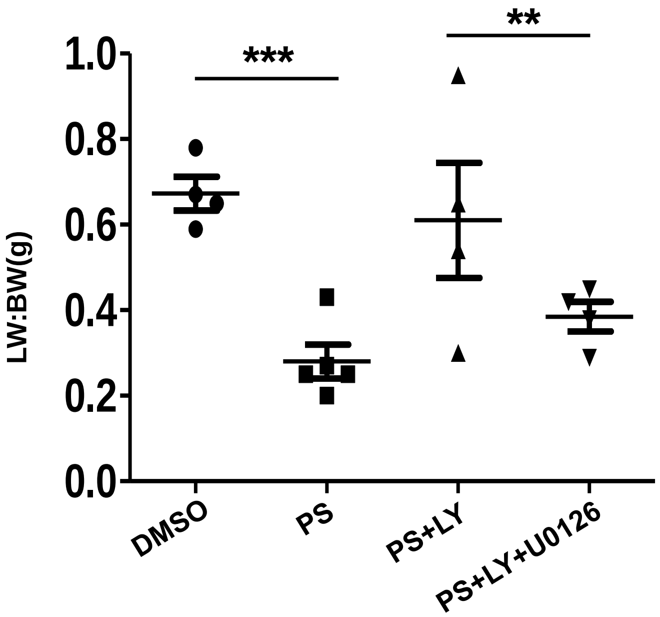

Figure S10. LW/BWs from each mouse bearing intravenously inoculated suspended LLC cells that were treated with DMSO, PS, PS+LY, or PS+LY+U0126 as described in Fig 8A.
